# INtervention for Cognitive Reserve Enhancement in delaying the onset of Alzheimer’s Symptomatic Expression (INCREASE), a randomized controlled trial: rationale, study design, and protocol

**DOI:** 10.1186/s13063-019-3993-0

**Published:** 2019-12-30

**Authors:** Daniela C. Moga, Brooke F. Beech, Erin L. Abner, Frederick A. Schmitt, Riham H. El Khouli, Ashley I. Martinez, Lynne Eckmann, Mark Huffmyer, Rosmy George, Gregory A. Jicha

**Affiliations:** 1 Department of Pharmacy Practice and Science, College of Pharmacy, University of Kentucky, Lexington, KY USA; 2 Department of Epidemiology, College of Public Health, University of Kentucky, Lexington, KY USA; 3 Sanders-Brown Center on Aging, Lexington, KY USA; 4 Department of Neurology, College of Medicine, University of Kentucky, Lexington, KY USA; 5 Department of Radiology, College of Medicine, University of Kentucky, Lexington, KY USA; 6 PRO2RX LLC Pharmacy Consulting Services, Lexington, KY USA

**Keywords:** Beers criteria, Inappropriate medication, Deprescribing, Comprehensive medication review, Medication therapy management, Interdisciplinary, Patient-centered, Cognitive reserve, Alzheimer’s disease, Dementia

## Abstract

**Background:**

The course of Alzheimer’s disease (AD) includes a 10–20-year preclinical period with progressive accumulation of amyloid β (Aβ) plaques and neurofibrillary tangles in the absence of symptomatic cognitive or functional decline. The duration of this preclinical stage in part depends on the rate of pathologic progression, which is offset by compensatory mechanisms, referred to as cognitive reserve (CR). Comorbid medical conditions, psychosocial stressors, and inappropriate medication use may lower CR, hastening the onset of symptomatic AD. Here, we describe a randomized controlled trial (RCT) designed to test the efficacy of a medication therapy management (MTM) intervention to reduce inappropriate medication use, bolster cognitive reserve, and ultimately delay symptomatic AD.

**Methods/design:**

Our study aims to enroll 90 non-demented community-dwelling adults ≥ 65 years of age. Participants will undergo positron emission tomography (PET) scans, measuring Aβ levels using standardized uptake value ratios (SUVr). Participants will be randomly assigned to MTM intervention or control, stratified by Aβ levels, and followed for 12 months via in-person and telephone visits. Outcomes of interest include: (1) medication appropriateness (measured with the Medication Appropriateness Index (MAI)); (2) scores from Trail Making Test B (TMTB), Montreal Cognitive Assessment (MoCA), and California Verbal Learning Test (CVLT); (3) perceived health status (measured with the SF-36). We will also evaluate pre- to post-intervention change in: (1) use of inappropriate medications as measured by MAI; 2) CR Change Score (CRCS), defined as the difference in scopolamine-challenged vs unchallenged cognitive scores at baseline and follow-up. Baseline Aβ SUVr will be used to examine the relative impact of preclinical AD (pAD) pathology on CRCS, as well as the interplay of amyloid burden with inappropriate medication use.

**Discussion:**

This manuscript describes the protocol of INCREASE (“INtervention for Cognitive Reserve Enhancement in delaying the onset of Alzheimer’s Symptomatic Expression”): a randomized controlled trial that investigates the impact of deprescribing inappropriate medications and optimizing medication regimens on potentially delaying the onset of symptomatic AD and AD-related dementias.

**Trial registration:**

ClinicalTrials.gov, NCT02849639. Registered on 29 July 2016.

## Background

Alzheimer’s disease (AD) is an important public health issue. Approximately 5.8 million Americans are currently living with AD [1], and this number is predicted to nearly triple by 2050 [2]. To forestall an impending crisis, the 2015 National Alzheimer’s Project Act (NAPA) report emphasized the need to identify effective prevention strategies to delay onset of symptomatic AD [3]. The biological disease course of AD has been elucidated, described as a 10–20 year preclinical period with progressive accumulation of amyloid plaques and neurofibrillary tangles, in the absence of symptomatic cognitive or functional decline [4]. The duration of this period is theoretically dependent on the rate of pathologic progression offset by compensatory mechanisms, collectively referred to as cognitive reserve (CR) [5]. Previous research has validated the importance of building and preserving CR to prolong this asymptomatic phase [5–7]. While much emphasis has been placed on developing and testing disease-modifying strategies targeting this preclinical phase of AD (pAD), little emphasis has been placed on currently available strategies targeting CR during pAD that may delay progression to the symptomatic stage of disease. Interventions designed to bolster CR (including aerobic exercise [8, 9], complex gameplay [10–12], diet [7, 13–16], and pharmacological interventions [17, 18]) have shown promise, but have not been proven to delay onset of symptomatic AD [5].

Medication therapy is a fundamental component of clinical care in older adults, but evidence suggests that pharmacotherapy in this population is often inappropriate [19]. Prescribing for older patients can be challenging due to factors such as age-related changes in adverse effect profiles and drug metabolism/catabolism, as well as the extensive use of polypharmacy to address multimorbidity [20, 21]. Lau et al. investigated medication use in older adults followed by National Institute on Aging-funded Alzheimer’s Disease Centers between 2005 and 2007, and estimated that 20% of those without dementia and 15% of those with dementia reported using at least one potentially inappropriate medication (PIM) as defined by the 2003 Beers criteria [19]. Another study found that frail elderly patients took an average of 15 medications (range 6 to 28) and experienced an average of 8.9 drug-related problems per patient (range 3 to 19), including inappropriate medication use and misuse and drug–drug and drug–disease interactions [22]. While these studies investigated PIM use on small, closely followed cohorts of patients, PIM use among older adults is widespread. A recently published paper used a nationally representative sample of the US older adults population and determined that 42.6% of older adult medication users reported at least one PIM as defined by the 2012 Beers criteria between 2006 and 2010 [23].

**Fig. 1:**
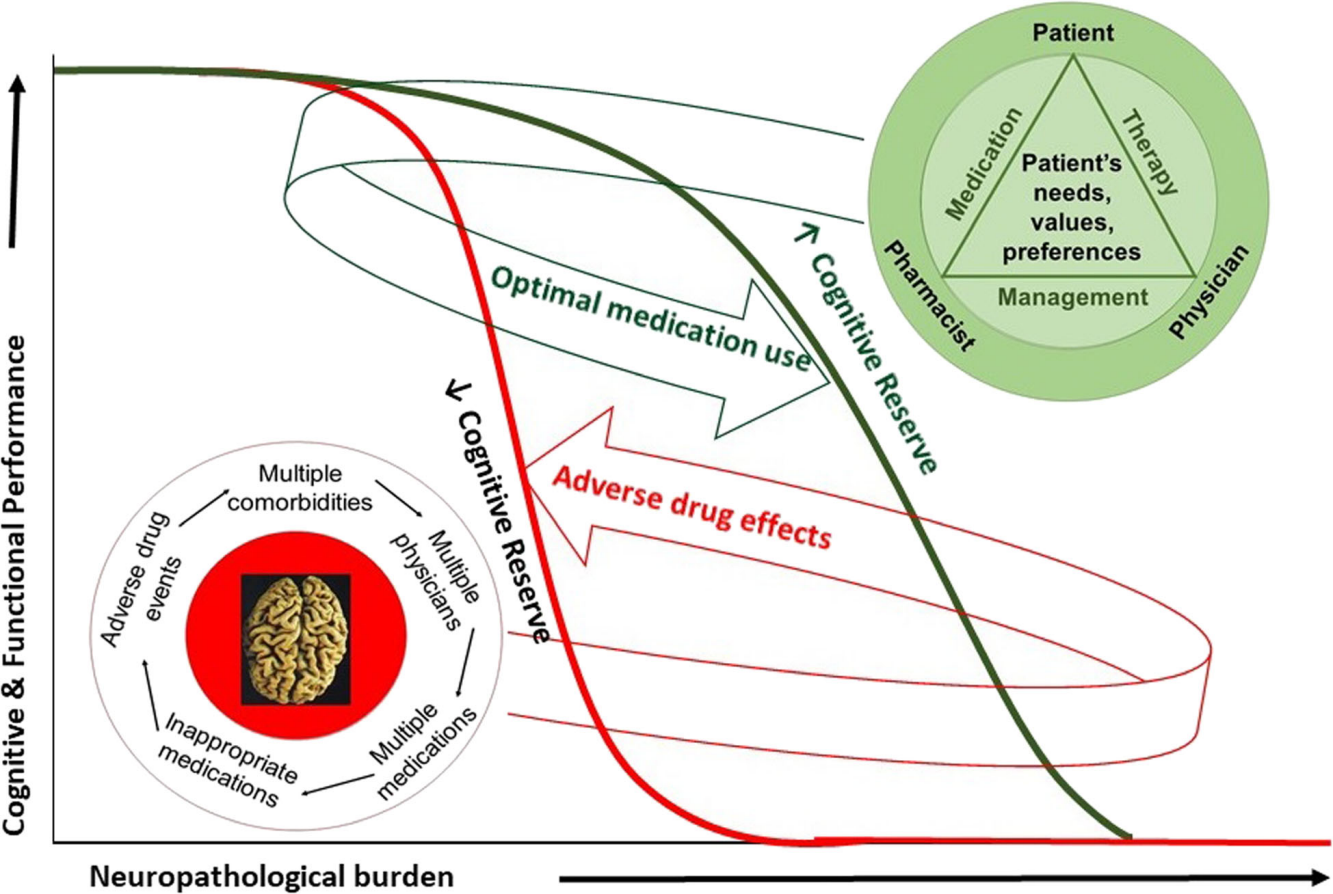
Patient-centered medication therapy management intervention to address the complexity of inappropriate medication use and bolster cognitive reserve

In a randomized controlled trial (RCT) using a patient-centered, clinician–pharmacist medication therapy management (MTM) intervention, we observed a 56% reduction in inappropriate anticholinergic drug use in older adults enrolled in the study [24]. These data document the efficacy of the MTM intervention to change inappropriate medication use and potentially delay dementia due to AD by maintaining CR. Based on our central hypothesis, as depicted in Fig. 1, interventions that deprescribe inappropriate medications and optimize treatment regimens for older adults with complex medical conditions may delay the substantial financial and societal impact of dementia due to AD by maintaining CR.

Here, we describe an RCT designed to expand the scope of the aforementioned pilot MTM intervention, from targeting only anticholinergic medications to targeting reduction in all inappropriate medications, bolster CR, and ultimately delay symptomatic AD. Finding the right balance between concomitantly treating several chronic conditions and avoiding medication-related negative effects is an important objective for healthcare providers, yet one that might be difficult to achieve [21, 25]. While clinicians regularly monitor and alter medication regimens when risks appear to outweigh benefits, the impact of inappropriate medication use is often not recognized by many healthcare providers. Interdisciplinary team approaches that focus on thorough medication reviews are designed to address this issue directly. The addition of a clinical pharmacist with extensive experience in conducting MTM reviews in older patients adds value for the brain health care provided to elderly at risk for PIM use. Our experience suggests that optimal medication management can be achieved through the development of a therapeutic triad including the clinician, pharmacist, and patient as outlined in Fig. 1.

### Objectives

The INtervention for Cognitive Reserve Enhancement in delaying the onset of Alzheimer’s Symptomatic Expression (INCREASE) study is designed to address two specific aims. The first aim is to conduct a 12-month RCT to evaluate the impact of a patient-centered, pharmacist–clinician team MTM intervention in deprescribing unnecessary and inappropriate medication, and thus limiting their use. The second aim evaluates the association of amyloid burden with CR dysfunction (measured as cognitive reserve change score (CRCS)) to evaluate the efficacy of delaying symptomatic disease progression. We hypothesize that higher amyloid burden reduces CR, thus increasing susceptibility to “unmasking” of cognitive impairment by environmental stressors such as inappropriate medication use, which may hasten the onset of clinically evident cognitive impairment and dementia. Our objectives are as follows:

1. Assess the effectiveness of the MTM intervention in reducing inappropriate medication use over the study period as determined by the medication appropriateness index (MAI; Aim 1) [26]
2. Investigate the association of β-amyloid positron emission tomography (Aβ-PET) and MAI with CR, operationalized as CRCS = scopolamine challenged cognitive test performance versus unchallenged performance (Aims 1 and 2)
3. Investigate the effects of the MTM and changes in MAI on CRCS in participants that are Aβ-PET positive or negative over the one-year study period (Aims 1 and 2)

## Methods/design

### Study design synopsis

The INCREASE study is a single-site, randomized, placebo-controlled clinical trial currently being conducted at the University of Kentucky (UK). We plan to enroll 90 non-demented older adults. Participants will be randomly assigned to treatment with the MTM intervention or to continue with standard of care procedures. After enrollment, all participants are followed for 12 months. The UK Institutional Review Board (IRB) approved all study procedures and all participants provide informed consent to participate (see Additional file 1). In addition, the study is also monitored by an independent Data Safety and Monitoring Board (DSMB) consisting of a geriatrician, a geriatric pharmacist, and a trial statistician, as well as representatives from the National Institute on Aging. The DSMB meets every 6 months to review the progress of the study and evaluate participants’ safety. All serious adverse events are reported to the IRB and the DSMB within 24 h. Before implementation, protocol modifications are approved by the UK IRB, and then reported to the DSMB and ClinicalTrials.gov.

### Study participants

Participants will be recruited from Lexington, KY and the surrounding area, using IRB-approved media outlets, outreach activities, physicians engaged in the memory disorders clinic, and community physician or personal referrals. The study cohort consists of non-demented, community dwelling older adults (aged 65 years and over) who regularly take at least one medication included on the Beers 2015 list. The complete list of inclusion and exclusion criteria is detailed in Table 1.

**Table 1:** INCREASE study eligibility criteria

Inclusion criteria	Exclusion criteria
1. Age ≥ 65 years	1. Allergy or known intolerance to scopolamine patches
2. Non-demented	2. Narrow-angle glaucoma
3. No previous reaction or contraindication to scopolamine patch, or medical condition warranting dose adjustment in scopolamine patch including but not limited to: open angle glaucoma, gastrointestinal or urinary outlet obstructions, seizures, or psychosis	3. Difficulty swallowing
4. No contraindications to Aβ PET scan including hypersensitivity to PET ligand (florbetapir) or radiation exposure in the past year that would exceed acceptable safe annual exposure in combination with the Aβ PET	4. Stomach or bowel problems (e.g., blockage, muscle weakness, ulcerative colitis)
5. Medically stable and able to complete all study activities, in the opinion of the investigator	5. Myasthenia gravis
6. Reporting at least one potentially inappropriate medication as listed in the Beers 2015 criteria	6. Blockage of the urinary tract
7. Living in the community	7. Seizures
8. Able to identify a study partner who will drive the participant to and from the scopolamine-challenged visits	8. Psychosis
9. Willing to participate in this intervention study	9. Contraindications to Aβ PET scan including hypersensitivity to PET ligand (florbetapir) or radiation exposure in the past year that would exceed acceptable safe annual exposure in combination with the amyloid β PET

*PET* positron emission tomography, *Aβ* amyloid beta

### Data collection and study procedures

**Fig. 2:**
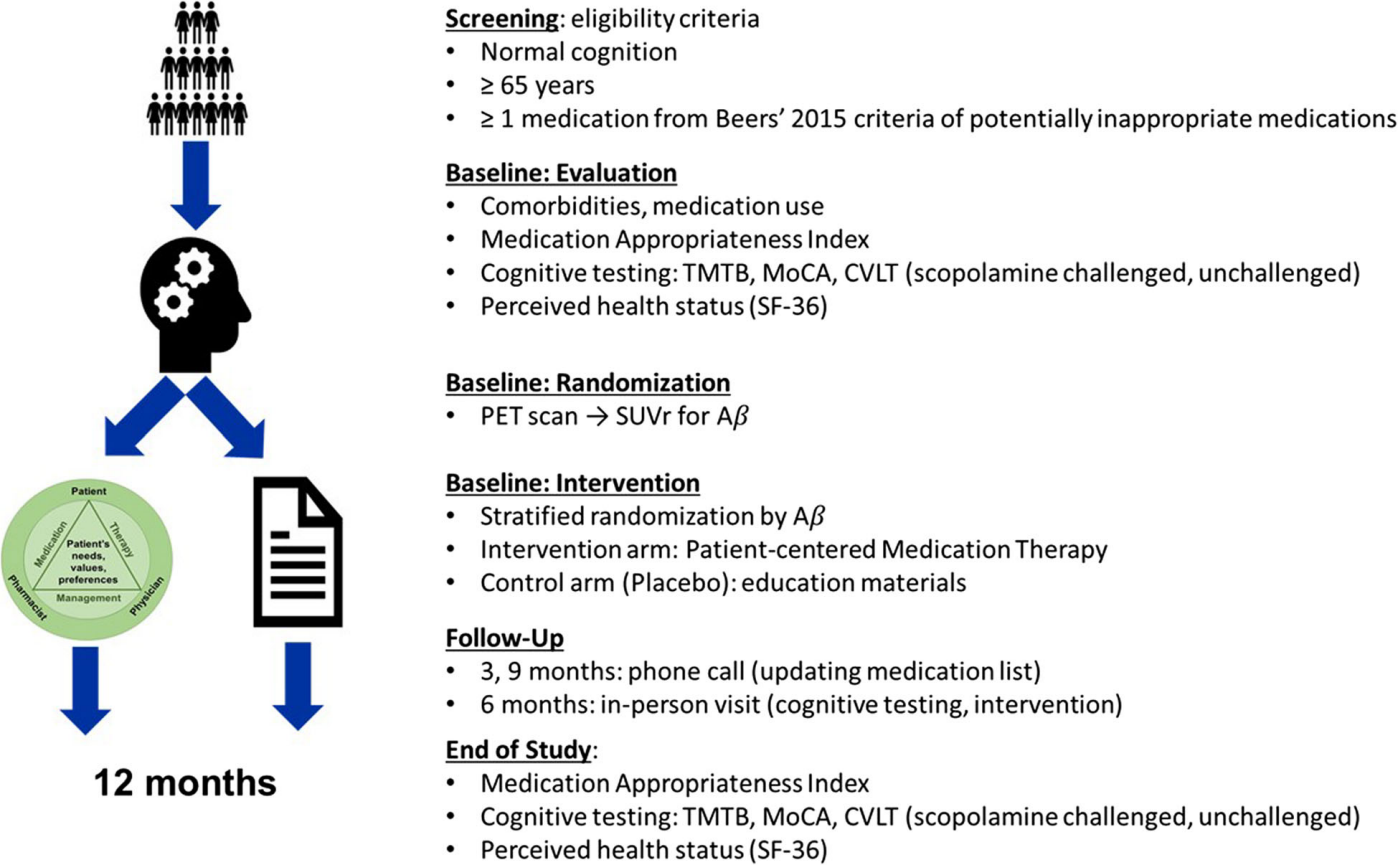
INCREASE study procedures. *TMTB* Trail Making Test B, *MoCA* Montreal Cognitive Assessment, *CVLT* California Verbal Learning Test, *PET* positron emission tomography, *Aβ* amyloid beta, *SUVr* standardized uptake value ratios

Data collected from study participants are captured on paper source documents and entered and managed using electronic data capture tools hosted at UK [27, 28]. Research Electronic Data Capture (REDCap) is a secure, web-based software platform designed to support data capture for research studies, providing 1) an intuitive interface for validated data capture; 2) audit trails for tracking data manipulation and export procedures; 3) automated export procedures for seamless data downloads to common statistical packages; and 4) procedures for data integration and interoperability with external sources. Study procedures are summarized in Fig. 2 and described in detail below.

### Scopolamine challenge

Scopolamine patches are a widely used, safe, effective, Food and Drug Administration (FDA)-approved therapy for prevention of emesis and motion sickness in adults. FDA-approved dosing indicates a dose of 1.5 mg patch every 3 days. In the INCREASE study, patches are used for a more limited period of time, overnight prior to challenge cognitive testing (≥ 4 h) to allow 90% steady state blood levels of approximately 87 pg/mL for free scopolamine and 354 pg/mL for total scopolamine. The patches are removed immediately after testing is complete. Following patch removal, plasma levels decline in a log-linear fashion with an observed half-life of 9.5 h and normal cholinergic function is generally regained within 24 h of patch removal.

Scopolamine challenge has been used to assess cognitive vulnerability (i.e., CR) in published studies and has been shown to influence cognitive test performance at comparable subcutaneous (SQ) doses [29]. In addition, the patch is an easier and more tolerable delivery method than SQ dosing, which would require prolonged clinic visits (> 3 h to reach steady state with SQ dosing that could only be reliably administered in clinic).

The concept of the CRCS is designed to operationalize cognitive reserve as a quantifiable, objective score that can be compared over time irrespective of, but in addition to, longitudinal change in unchallenged cognitive test performance. We acknowledge that this calculation has not been utilized previously in studies but can be deduced from published data on scopolamine challenge in other paradigms as challenged vs unchallenged cognitive test scores. A difference equal to 0 would indicate healthy cognitive reserve reflective of the participant’s ability to tolerate the patch without diminution in cognitive test scores, whereas change scores lower than 0 (and in excess of measurement error) would indicate anticholinergic sensitivity as a surrogate quantitative measure of cognitive reserve [29].

### Randomization and blinding

Randomization is stratified by quantitative Aβ standardized uptake values ratios (SUVr), normalized to cerebellum, as determined following the PET scan: SUVr < 1.2, 1.2 ≤ SUVr < 1.4, and SUVr ≥ 1.4 [30, 31]. Utilization of these three strata (each stratum includes approximately one-third of the study population) ensures that amyloid burden will be equally distributed across the treatment groups. Within SUVr strata, participants are randomized to the MTM intervention or standard of care with equal probability. Randomization occurs on the third study visit (Table 2). Consecutive treatment assignments are sealed in opaque envelopes by the study statistician and opened by the study coordinator following the screening visit, PET scan, and baseline cognitive assessments.

**Table 2:** Overview of study procedures

Procedure	Screening	Baseline cognitive testing		Month 3	Month 6	Month 9	End-of-study cognitive testing	
		Scopolamine challenged	Non-challenged				Scopolamine challenged	Non-challenged
Study week	−5 ± 2 weeks	− 4 ± 1	0 ± 1	13 ± 1	26 ± 1	39 ± 1	52 ± 1	56 ± 1
Demographics	X							
Health history	X	X	X	X	X	X	X	X
Medication review	X	X	X	X	X	X	X	X
NAART	X							
TMTB		X	X				X	X
CVLT		X	X				X	X
MoCA		X	X				X	X
SF-36	X							X
ECG	X							
Physical exam	X							X
Neurological exam	X							X
Gait and balance	X	X			X		X	X
Aβ-PET imaging	X							
MTM intervention			X		X		X	
Telephone follow-up				X		X		

*NAART* North American Adult Reading Test, *TMTB* Trail Making Test B, *CVLT* California Verbal Learning Test, *MoCA* Montreal Cognitive Assessment, *SF-36* Short-Form 36, *ECG* electrocardiogram, *Aβ* amyloid beta, *PET* positron emission tomography, *MTM* medication therapy management

### PET scan

Aβ-PET imaging is currently FDA-approved for the detection of cerebral amyloid deposition in patients with dementia. It does not currently have an indication for the detection of cerebral amyloidosis in patients that do not have dementia. With these caveats in mind, Aβ-PET imaging has been reliably used to detect pAD across numerous studies, detecting cerebral amyloidosis in approximately one-third of the cognitively normal population over the age of 65 years [4, 32]. Current clinical use of Aβ-PET scans relies on a subjective determination of dichotomous presence vs absence of radiolabel binding to cortical regions, which is subject to multiple sources of error, including visual color discrimination and the experience of the interpreting radiologist. Quantitative SUVR determinations are more precise and allow for the specification of amyloid burden as a continuous variable that will be leveraged in the current study allowing associations with cognitive test performance and CRCS to be examined secondarily across the continuum of cerebral amyloid burden that characterizes pAD [33].

For each scan, the participant receives a single intravenous administration of approximately 370 MBq (10 mCi) of florbetapir F 18 (fast intravenous push). The injection of the imaging agent is followed by a saline flush. After an uptake period of 50 min, participants will be positioned in a head stabilization unit designed for PET/CT scanners. All PET/CT scans are performed on a Siemens Biograph TruePoint 6-slice (Siemens Healthcare, Erlangen, Germany). Low resolution CT images of the head will be acquired (120 kVp, FOV 50 cm, pitch 0.55, 0.5 s rotation time, slice thickness 4 mm, care dose). PET images of the brain are then collected for a 20-min, 3D emission scan. The emission images will be reconstructed using 256×256 matrix and all pass filter. Injected dose, time of injection, residual radiotracer within the syringe after injection, and patient weight and height are recorded to be used for the standardized uptake value calculation based on the following equation:

$$ SUV=\frac{\mathrm{uptake}\ \left(\mathrm{kBq}/\mathrm{mL}\right)}{\mathrm{injected}\ \mathrm{dose}\ \left(\mathrm{kBq}\right)/\mathrm{patient}\ \mathrm{weight}\ \left(\mathrm{g}\right)\ } $$

Image analysis will be done using dedicated PET/CT image analysis software, Mirada (version XD3, Mirada Medical Ltd, New Road, Oxford, UK). Multi-planar reconstruction (MPR) of the axial images of both PET and CT datasets as well PET/CT fused images are reconstructed in the sagittal and coronal planes. Change in brain amyloid burden (as assessed by florbetapir binding and measured by mean cortical standardized uptake value ratio (SUVr)) will be analyzed. Total brain SUVr is calculated as described previously by the AV45-A11 Study Group [33]. Specifically, multiple volumes of interests (VOI) will be placed on different parts of the cortex (frontal, parietal, temporal, occipital, precuneus, posterior cingulate regions) as well as the pons and cerebellum. SUV ratios for each VOI will be generated using the cerebellum and pons (SUVr denominators).

### Study intervention

As the MTM intervention is educational and delivered in person, complete blinding of treatment assignment is not possible. However, we are taking the following steps to minimize potential bias and achieve the maximum level of blinding possible by this design: (1) when reviewing the medication list prior to the intervention, the study coordinator, study pharmacist, and clinician are unaware of the group allocation; (2) data analysis will be blinded to the intervention.

At enrollment, each participant is asked to bring all prescription and non-prescription medications and supplements they currently use. In a face-to-face interview, study personnel collect information on treatment indication, duration, dose and mode of administration, and adherence [34]. If participants report any adverse effects, study personnel record information on the suspected medication, symptomatology, and subsequent treatment modification (whether initiated by the participant or a healthcare provider) [34].

The study pharmacist then reviews the information and prepares prioritized written recommendations as follows: (1) a list of potentially inappropriate medications taken by the patients that are included in, but are not limited to, the 2015 Beers criteria [35] (the “problem list”); (2) a proposed action for each medication in the problem list (discontinuation, treatment modification (including suggested alternative or dose change), or treatment continuation when medically necessary); and (3) proposed action for any other prescription or non-prescription medication or supplement taken by the participant that might be inappropriate and/or unnecessary. Where appropriate, the proposed alternatives include medications suggested in the 2015 Beers 2015 criteria as “Alternative medications for medications in the use of high-risk medications in the elderly and potentially harmful drug-disease interactions in the elderly quality measures” [35, 36]. All study participants, regardless of their group assignment, are provided with educational materials focused on appropriate medication use and being an active participant in their healthcare team: “Avoiding overmedication and harmful drug reactions” (www.HealthinAging.org), “Ten medications older adults should avoid or use with caution” (www.HealthinAging.org), and “Be an active member of your health care team” (https://www.fda.gov/drugs/resources-you/be-active-member-your-health-care-team-article).

Throughout study follow-up, the control group receives standard medical care from their primary care providers. The MTM intervention is based on patient-centered principles by addressing the specific needs of each individual patient, taking into consideration the individual patient’s preferences and values, and by empowering the patient to take responsibility and fully participate in the decision-making process as an equal team player [37–39]. Following the preliminary medication review described above and the communication between the pharmacist and the clinician, those randomized to the MTM intervention meet with the pharmacist–clinician team during case conferences to discuss the problem list and decide on final recommendations for discontinuation or change related to inappropriate medications. The final recommendations and their rationale, along with general medication information, are discussed by the team with the participant. For the participants included in the MTM intervention group, the written recommendations and proposed changes are shared with their primary care providers, who are consulted on the best approach to improve outcomes. Although recommendations are made, the study team cannot force changes. For the duration of the study, the study coordinator contacts the participant every 3 months to follow-up on the proposed changes during the MTM intervention and to determine the need for additional pharmacist evaluations of newly prescribed medications.

**Fig. 3:**
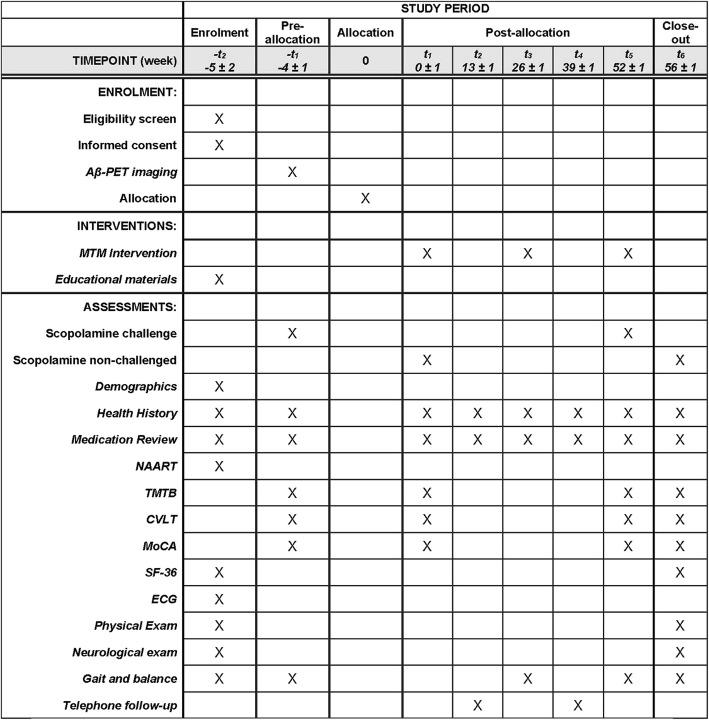
INCREASE study schedule of enrolment, interventions, and assessments (SPIRIT figure). *NAART* North American Adult Reading Test, *TMTB* Trail Making Test B, *CVLT* California Verbal Learning Test, *MoCA* Montreal Cognitive Assessment, *SF-36* short-form 36, *ECG* electrocardiogram, *Aβ* amyloid beta, *PET* positron emission tomography, *MTM* medication therapy management

The detailed schedule of study procedures by specific visits is included in Table 2 and Fig. 3.

## Study outcomes

Study outcomes are summarized in Table 3 and described in detail below.

**Table 3:** Study outcomes

Study outcomes			
Primary	Medication appropriateness	MAI	Change from pre- to post-intervention
	Executive function	TMTB	CRCS: difference in scopolamine-challenged and unchallenged z-scores
Secondary	Global cognition	MoCA	
	Memory	CVLT	
	Perceived health status	SF-36	Change from pre- to post-intervention

*MAI* medication appropriateness index, *TMTB* Trail Making Test B, *CVLT* California Verbal Learning Test, *MoCA* Montreal Cognitive Assessment, *SF-36* Short-Form 36

### Medication Appropriateness Index (MAI)

The MAI provides explicit instructions and examples to guide rating of medications as “appropriate”, “marginally appropriate”, or “inappropriate” based on ten criteria [26]. MAI assessments made by a clinical pharmacist and a physician demonstrated high inter- and intra-rater reliability (kappa = 0.83 and 0.92, respectively) [26]. While our focus is on medications considered potentially inappropriate by the 2015 Beers criteria, all medications reported by the participants at baseline and follow-up visits are evaluated by the study pharmacist in collaboration with the study clinician. Medication appropriateness is assessed by the study pharmacist in a blinded manner, prior to randomization for the baseline assessment, and without knowledge of the group assignment at the end of the study visit. We will also measure the reduction in the number of PIMs from baseline to the end of the study.

### Cognitive reserve

We operationalize a residual measure of CR utilizing sequential cognitive testing under a scopolamine challenge, followed after 4 weeks by unchallenged cognitive testing, as described in the Scopolamine challenge section. The difference in challenged and unchallenged performance is calculated as the cognitive reserve change score (CRCS) at baseline and the end of study (EOS). This novel method of operationalizing a cognitive reserve measure with an anticholinergic challenge has the added benefit of minimizing learning effects that may arise as a result of the sequential cognitive testing.

Several cognitive measures, including Trail Making Test B (TMTB) [40, 41], Montreal Cognitive Assessment (MoCA) [42, 43], and California Verbal Learning Test (CVLT) [44], will be used when calculating CR, each highlighting different components of cognition. All the tests are also available in multiple validated test versions, which can limit learning effects from repeated testing [45, 46]. TMTB was selected as the primary outcome measure based on our previously published work on inappropriate medication use in older adults [35, 47]. Our statistical considerations and power analysis are based on the data from this work. TMTB measures higher order executive function, which is a prime target for cognitive changes resulting from PIM use [35, 47], and has been shown to be a sensitive measure of cognitive decline in pAD [48]. MoCA and CVLT were chosen as global cognition and memory measures (respectively) to allow comparison to other cohorts, including the longitudinal cohort at the Alzheimer’s Disease Center at University of Kentucky (UK-ADC) [42, 43].

### Perceived health status

Change in perceived health status is measured to estimate the overall impact of the MTM intervention using the Short Form Health Survey (SF-36) at baseline and EOS. The SF-36 is a validated generic instrument that evaluates eight health concepts categorized into three major health attributes: (1) functional status (i.e., physical functioning, social functioning, role limitations due to physical problems, role limitations due to emotional problems); (2) well-being (mental health, vitality, bodily pain); and (3) general health perception (an overall evaluation of health) [49]. Previous research has reported that the SF-36 correlates with Sickness Impact Profile scores, a more thorough health status evaluation that could not be conducted feasibly in this trial [50, 51].

## Sample size

As noted previously, the study will enroll 90 participants. We estimate that approximately 30 participants will have elevated Aβ levels (SUVr ≥ 1.4), with the remaining two-thirds split between the lower SUVr strata described above.

Based on our current and previous studies using MAI as the primary outcome, we calculated the sample size to detect a clinically relevant mean difference of 1.0 between baseline and follow-up assessments for the intervention group vs no change in the control group. We will need 17 participants in each group to detect this difference with 80% power at a significance level (α) of 0.05. As we expect the effect of the intervention to be even greater for those with pAD, we have based the power analysis at a mean difference of 1.5 in MAI. Based on this assumption, for the subgroup analysis of subjects with pAD, we would need nine participants per group to detect this difference with 80% power at α = 0.05, and 11 per group to detect this difference with 90% power. These MAI effect sizes are rather conservative as previous studies show that medication reconciliation interventions can determine a mean MAI change ranging between 1.9 and 17 [52].

Our previous studies have demonstrated that cognitively intact older adults who used anticholinergic medications did not show learning effects for TMTB (i.e., scores remained unchanged over time), while nonusers showed a statistically significant improvement after 6 years [53]. Our findings are consistent with another study in younger adults that identified reduced psychomotor and executive function in participants treated with 0.2 mg scopolamine subcutaneously (− 0.75 and − 0.50 standard deviations, respectively) [54]. Since TMTB involves both psychomotor and executive function, it is reasonable to assume that we will observe deficits on this instrument in non-demented older adult participants challenged with scopolamine. Thus, we will compute age- and education-adjusted TMTB z-scores based on normative data for cognitively intact older adults [41]. Assuming our scopolamine challenge will induce deficits in cognitively intact older adults at least at the levels reported in younger adults, we will need 32 participants per group to detect a 0.50 SD improvement in the CRCS z-score with 80% power at α = 0.05, and 14 per group to detect a 0.75 SD improvement with 80% power in participants with pAD.

## Analysis strategy

To examine the effect of the intervention on medication appropriateness, we will perform analysis of covariance (ANCOVA), with the dependent variables being the difference between outcome measures at baseline and EOS, and the baseline measurement included as a covariate. Because the SF-36 does not produce a single overall measure and to reduce the number of comparisons, multivariate analysis of covariance (MANCOVA) will be used to simultaneously estimate the effect of the MTM intervention on the eight SF-36 health concepts. There are no planned interim analyses for INCREASE. Adverse events related to study participation are monitored by the UK IRB and the study DSMB and reported according to Federal regulation. At the minimum, the DSMB meeting is every 6 months.

## Discussion

The current paper describes the rationale and protocol of the INCREASE study, a single-center RCT designed to investigate (1) the impact of a patient-centered, pharmacist-clinician team MTM intervention in reducing unnecessary and inappropriate medication use, and (2) the interplay of deprescribing inappropriate medication and amyloid burden on cognitive reserve deficits and decline. Combining state-of-the-art detection of pAD through Aβ-PET with a unique patient-centered interdisciplinary MTM intervention is highly innovative, and directly translatable to clinical practice.

We hypothesize that the burden of pAD pathology will dampen CR, increasing susceptibility to “unmasking” of dementia symptoms by environmental stressors such as inappropriate medication use. In addition, by reducing CR, inappropriate medication use may hasten the onset of clinically evident dementia, prolonging the symptomatic phase of disease where health care costs are maximally incurred. Our preliminary work demonstrates that such impact is both feasible and achievable through practical establishment of procedures and policies designed to evaluate and reduce inappropriate medication use in older adults. The INCREASE study directly addresses a fundamental gap in existing knowledge on how MTM interventions may prove beneficial in delaying AD onset, shortening the overall duration of symptomatic disease expression, and reducing healthcare costs.

Some potential limitations of the current study lie in the use of the CRCS measure, which has not been previously validated. This concept, however, is based on scientific data that demonstrate that such a measure exists [29], that it can be calculated easily, and that such an approach can move to the field of intervention in pAD forward [32]. Furthermore, its calculation is in line with other methods being developed to operationalize CR [55]. Regardless, the INCREASE study is strengthened by preliminary interventional data demonstrating that our primary outcome measure of MAI change is achievable within the proposed study design. The potential economic health impact of the study warrants exploration of this approach to the preclinical phase of AD and reducing the duration of symptomatic disease, where most health expenditures lie.

At the time of writing, recruitment and enrollment are ongoing and are expected to be completed by early 2020. Follow-up measures will be completed by early 2021 with results expected to become available in late 2021.

## Trial status

- Current protocol version number and date: version 6, dated August 27, 2019
- Date recruitment began: March 23, 2017
- Approximate date when recruitment will be completed: March 31, 2020

## Supplementary information

**Additional file 1. **SPIRIT 2013 checklist.

## Data Availability

In accord with Federal regulations, this trial is registered with ClinicalTrials.gov and all raw data, stripped of identifiers, will be made available to interested parties and researchers with submission of written request to the study PI, not sooner than one year after acceptance and publication of the primary manuscript. There will be no charge for release of the dataset.

## Abbreviations

AD: Alzheimer’s disease
ANCOVA: Analysis of covariance
Aβ: Amyloid β
CR: Cognitive reserve
CRCS: Cognitive Reserve Change Score
CT: Computed tomography
CVLT: California Verbal Learning Test
ECG: Electrocardiogram
FDA: Food and Drug Administration
INCREASE: INtervention for Cognitive Reserve Enhancement for delaying the onset of Alzheimer’s Symptomatic Expression
IRB: Institutional review board
MAI: Medication Appropriateness Index
MoCA: Montreal Cognitive Assessment
MPR: Multi-planar reconstruction
MTM: Medication therapy management
NAART: North American Adult Reading Test
NAPA: National Alzheimer’s Project Act
pAD: Preclinical Alzheimer’s disease
PET: Positron emission tomography
PIM: Potentially inappropriate medications
RCT: Randomized controlled trial
REDCap: Research electronic data capture
SD: Standard deviation
SF-36: Short Form-36
SUVr: Standardized uptake value ratio
TMTB: Trail Making Test B
